# Engineering the expression of plant secondary metabolites-genistein and scutellarin through an efficient transient production platform in *Nicotiana benthamiana* L.

**DOI:** 10.3389/fpls.2022.994792

**Published:** 2022-09-06

**Authors:** Xinghao Yao, Kuanyu Wuzhang, Bowen Peng, Tiantian Chen, Yaojie Zhang, Hang Liu, Ling Li, Xueqing Fu, Kexuan Tang

**Affiliations:** Joint International Research Laboratory of Metabolic and Developmental Sciences, Frontiers Science Center for Transformative Molecules, Plant Biotechnology Research Center, Fudan-SJTU-Nottingham Plant Biotechnology R&D Center, School of Agriculture and Biology, Shanghai Jiao Tong University, Shanghai, China

**Keywords:** plant natural products, transient production platform, scutellarin, genistein, synthetic biology, *Nicotiana benthamiana*

## Abstract

Plant natural products (PNPs) are active substances indispensable to human health with a wide range of medical and commercial applications. However, excessive population growth, overexploitation of natural resources, and expensive total chemical synthesis have led to recurrent supply shortages. Despite the fact that the microbial production platform solved these challenges, the platform still has drawbacks such as environmental pollution, high costs, and non-green production. In this study, an efficient platform for the production of PNPs based on the transient expression system of *Nicotiana benthamiana* L. combined with synthetic biology strategies was developed. Subsequently, the feasibility of the platform was verified by a simple “test unit.” This platform was used to synthesize two high-value PNPs: genistein (5.51 nmol g^–1^ FW) and scutellarin (11.35 nmol g^–1^ FW). Importantly, this is the first report on the synthesis of scutellarin in heterologous plants. The platform presented here will possibly be adopted for the heterologous production of genistein and scutellarin in tobacco plants as a novel and sustainable production strategy.

## Introduction

Plant natural products (PNPs) are plant secondary metabolites that have a wide range of bioactive properties, many of which are vital to humanity. To date, PNPs have been widely used in fragrances, natural dyes, dietary supplements, foods, cosmetics, and pharmaceutical industries (Birchfield and McIntosh, 2020). Based on their biosynthetic pathways, PNPs can be classified into three main groups: flavonoids, alkaloids, and terpenoids (Saxena et al., 2013). Genistein is one of the most abundant pharmacologically active flavonoids in soybean, and its biosynthesis pathway is well elucidated. In recent years, genistein has received a lot of attention for its potential to prevent osteoporosis (Marini et al., 2007), lower the risk and incidence of cancer and cardiovascular diseases, and ease menopausal symptoms (Nestel et al., 1999; Cotterchio et al., 2007; Marco et al., 2007). However, the content of genistein in soybean varies depending on the variety and growing conditions and/or environment (Eldridge and Kwolek, 1983). Like genistein, scutellarin is another major bioactive constituent of flavonoids extracted from *Erigeron breviscapus* (Asteraceae family) (Wang and Ma, 2018). Similarly, scutellarin has a number of pharmacological effects, including suppressing tumor growth and metastasis (Zeng et al., 2022), treating cardiovascular diseases (Prince et al., 2015), promoting blood circulation and dissolving stasis (Liu et al., 2010). Scutellarin’s efficacy and necessity for human health are undeniable, but its manufacture still predominantly depends on limited plant resources. Resources for *E. breviscapus* are currently scarce, and the pharmaceutical sector uses more than a million kilograms of the plant annually, drastically depleting its wild resources (Li et al., 2011). Additionally, more than 10 million people in China utilize scutellarin and associated medications each year, and the available supply of scutellarin can no longer keep up with the country’s expanding demand market (Zhang et al., 2013). Based on the health-promoting effects of these two types of PNPs, researchers have tried different production methods.

However, the use of total chemical synthesis is not economical due to its complex structure. Furthermore, although the microbial platform has successfully produced genistein and scutellarin (Horinouchi, 2009; Liu et al., 2018), the chassis still has several disadvantages (e.g., labor-intensive production, high costs, pollution of the environment, and poor tolerance of the products) (Reed et al., 2017; Shao et al., 2017). Therefore, the use of plant chassis to produce genistein and scutellarin is a promising approach, which will reduce the production cost of genistein and scutellarin, reduce the exploitation of plant resources, alleviate the supply pressure caused by rapid population growth, etc. In addition, it is also important to note that heterologous production of scutellarin in plants has not been reported previously.

*Nicotiana benthamiana* (*Nicotiana benthamiana* L.), a wild relative of tobacco originally from Australia, is peerless in its susceptibility to agroinfiltration and its amenability to transiently expressing transgenes (Horsch et al., 1985). As a common tool, the transient expression system of tobacco has been applied to produce a variety of PNPs (especially for the treatment of serious diseases). Examples include artemisinin (used for malaria treatment) and cannabinoids (used to prevent SARS-CoV-2 coronavirus from entering human cells) (Wang et al., 2016; Gülck et al., 2020; Van Breemen et al., 2022). Although the above cases demonstrated the feasibility of the traditional transient expression system of tobacco, it could not be applied to large-scale production. Therefore, it is urgent to develop a novel transient production platform. Up to now, little research in this area has been conducted.

As an interdisciplinary field, synthetic biology offers a strategy for the development of PNPs. First of all, fast and efficient methods for multigene assembly are essential for plant synthetic biology. The Golden Gate cloning (GGC), based on type IIS restriction endonuclease, is efficient and results in “scarless” assemblies (Engler et al., 2009). Secondly, the effect of untranslated regions (UTR) on protein expression has been widely demonstrated (Sainsbury and Lomonossoff, 2008). Combining these strategies with improved agroinfiltration techniques, Stephenson et al. (2018) produced gram-scale quantities of purified triterpene in just a few weeks. Thirdly, the production level of various PNPs is considerably increased by the use of transcriptional regulation by activating secondary metabolic pathway genes. Butelli et al. (2008) introduced *Delila* and *Rosea1* genes from *Antirrhinum majus* into tomato (*Solanum lycopersicum* L.), resulting in a deep purple fruit. This indicates that large amounts of anthocyanins were produced in the fruit, and at concentrations comparable to those found in blackberries and blueberries. Overexpression of maize *Lc* or *Arabidopsis PAP1* resulted in enhanced total flavonoid content in *Scutellaria baicalensis* hairy roots (up to 80.5 ± 6.15 mg g^–1^ DW and 133 ± 7.66 mg g^–1^ DW, respectively) (Chang et al., 2021). Unlike other transcription factors (TFs), AtMYB12 not only activates genes involved in flavonoid biosynthetic pathways, but also enhances primary metabolism, thereby increasing the supply of secondary metabolic precursors (Zhang et al., 2015). The application of the above technologies ensures the optimal yield of the target products, and the development of a vacuum infiltration device could also significantly increase the conversion efficiency of the leaves. Taken together, the combination of these synthetic biology strategies and the vacuum infiltration device constituted the efficient transient production platform (E-platform). However, the assembly of multi-gene pathways and heterologous synthesis (extraction, isolation, and identification) of the PNPs in plants are lengthy and complex processes. In order to reduce the time wasted due to failed experiments, it is essential to check the compatibility of the platform through testing sessions. The expression or non-expression of the two reporters in the “test unit” directly determines the applicability of the platform. One is enhanced green fluorescent protein (EGFP), which has been widely used as an indispensable imaging and tracking tool for various biological studies (Enterina et al., 2015). The other is SlAN2-Like, a key TF determining the intense purple skin of tomato, which has been shown to promote anthocyanidin accumulation (Sun et al., 2020). To determine whether the SlAN2-Like was functioning, the change in leaf color was examined.

Herein, we achieved the production of genistein and scutellarin in *N. benthamiana* by using synthetic biology strategies and developed a novel E-platform, which will provide theoretical support for the rapid and large-scale production of high-value PNPs and can be used as an alternative route for the extraction of PNPs from plants.

## Materials and methods

### Plant material and cultivation

*Nicotiana benthamiana* (GenBank: PRJNA170566) seeds were sown in the soil with a 1:1 ratio of peat (Pindstrup Horticulture Co., Ltd., Denmark) and vermiculite, and covered with a transparent plastic cover. After 1 week, the seedlings were transferred to an independent flowerpot (specifications: diameter 10 cm, height 8.5 cm), and agroinfiltration was carried out after 4 weeks. All growth periods of *N*. *benthamiana* were in the greenhouse with conditions as follows: temperature 25 ± 1°C, humidity 60%, light intensity 109 μmol photons m^–2^ s^–1^, and photoperiod of 16 h light/8 h dark.

### Acquisition of gene elements and assembly of expression cassette

The sequences of the Cauliflower mosaic virus 35S promoter (CaMV35S-P) and the modified 5′-UTR of CPMV RNA2 were cloned with special primers (containing *Bsa*I restriction sites and 4-nt overhangs) from pGREENII0800-LUC and pEAQ-HT-DEST vectors, respectively. Similarly, the “3′-UTR + nopaline synthase terminator (NOS-T)” sequence was also derived from the pEAQ-HT-DEST vector, which was kindly provided by Liu et al. (2020). Genes encoding AtMYB12 (At2g47460), GmIFS (AF195798), GmHID (AB154415), EbFNSII (KC521362), EbF6H (KU237240), and EbF7GAT (KU237242) were all codon-optimized toward *N. benthamiana* by Shenggong Bioengineering Co., Ltd., (Shanghai, China). In addition, *Thosea asigna* virus 2A (2A) was also synthesized in the aforementioned company.

**FIGURE 1:**
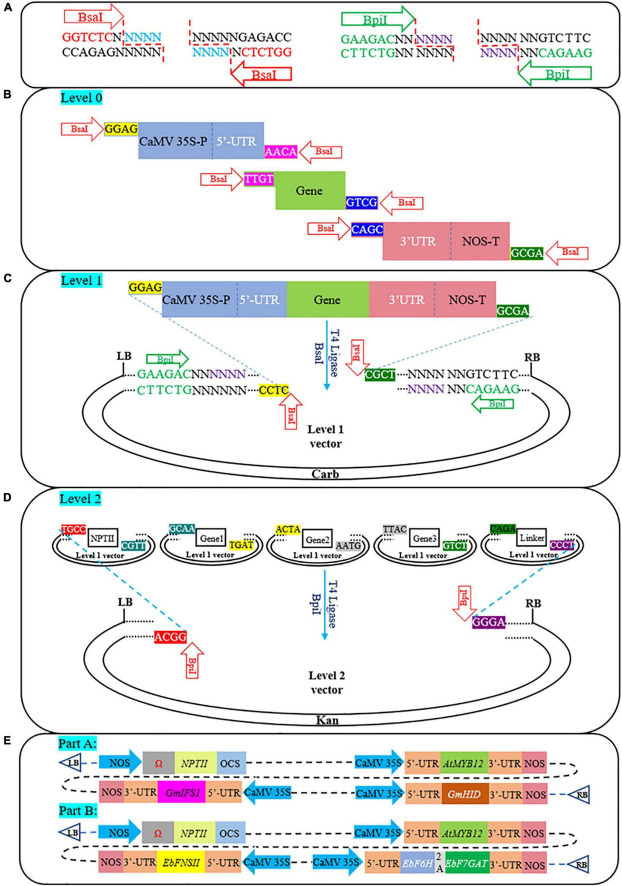
A schematic illustrating the assembly of multigene constructs based on the GGC. **(A)** Recognition sequence, cleavage site, and post-cleavage overhangs of two type IIS restriction enzymes (*Bsa*I and *Bpi*I). **(B)** The *Bsa*I recognition sites (GGTCTC) and specific fusion sites (e.g., GGAG and ACAA) on both sides of each Level-0 module (e.g., promoter, UTR, ORF, 2A, and terminator) were introduced by primers. **(C)** Level-1 module digestion-ligation process. Each expression cassette was assembled into Level-1 destination vectors. **(D)** The final Level-2 modules construction process. Multiple Level-1 modules formed the final vector via a one-step digestion-ligation reaction of *Bpi*I and T4 ligase. **(E)** Schematic diagram of T-DNA regions containing pathway genes involved in the synthesis of genistein and scutellarin. Part A, genistein; Part B, scutellarin; NPTII, aminoglycoside phosphotransferase from Tn5; Ω, tobacco mosaic virus 5′-leader sequence. 2A, *Thosea asigna* virus 2A.

GGC was used for expression cassette assembly, and the recognition sites and cleavage patterns of the two enzymes (*Bsa*I and *Bpi*I) involved in this process are shown in Figure 1A. All vectors and components used in this process were divided into three modules: Level-0 (Promoter/5′-UTR/ORF/2A/3′-UTR/Terminator), Level-1 (pICH47732/pICH47741/pICH47751/pICH47761/pICH41780), and Level-2 (pAGM4723) (Figures 1B–D). The aforementioned plasmids were gifted by Sylvestre Marillonnet (Weber et al., 2011). Except for the ORF (synthesized into the pUC57 vector), enzyme cleavage sites and specific fusion sites at both ends of each Level-0 module were introduced by primers, and the PCR products were linked into the pLB vector (TIANGEN Biotech, Beijing, China) (Figure 1B). It is worth noting that CaMV35S-P and 5′-UTR are two separate parts, and they need to be spliced before the assembly process starts. As shown in Supplementary Figure 1, CaMV35S-P and 5′-UTR were constructed into the pICH47751 vector. Subsequently, they were amplified with special primers to form the standard Level-0 module (Figure 1B). The construction process of multigene pathways is roughly divided into 2 steps (Figures 1C,D), and more details of the process and the PCR conditions are provided in Supplementary Table 1. Supplementary Table 3 shows all the primers used in this study.

### Preparation of agroinfiltration

Agroinfiltration of leaf tissue with binary vectors is also a rapid method of producing metabolites in plants. *Agrobacterium tumefaciens* strain GV3101 (pSoup-p19, encodes a viral suppressor of RNA silencing) was used for transient expression analysis of *N*. *benthamiana*. The agroinfiltration procedure was based on the method of Wood et al. (2009) with minor modifications. *A*. *tumefaciens* was grown overnight in LB medium (supplemented with 10 mM 2-(N-morpholino)ethanesulphonic acid (pH 5.6), 40 μM acetosyringone, 50 μg mL^–1^ kanamycin and 25 μg mL^–1^ rifampicin). The *A. tumefaciens* cells were centrifuged at 4,000 rpm for 10 min until they attained an optical density at 600 nm (OD_600_) between 1 and 1.5. The supernatant was discarded, and *A*. *tumefaciens* cultures were resuspended in the infiltration buffer (10 mM MgCl_2_, 200 μM acetosyringone) to OD_600_ = 0.8 and incubated for 3 h at room temperature. Additionally, it’s crucial to make a suitable bacterial suspension in accordance with production requirements.

### The operation mode of the novel E-platform

**FIGURE 2:**
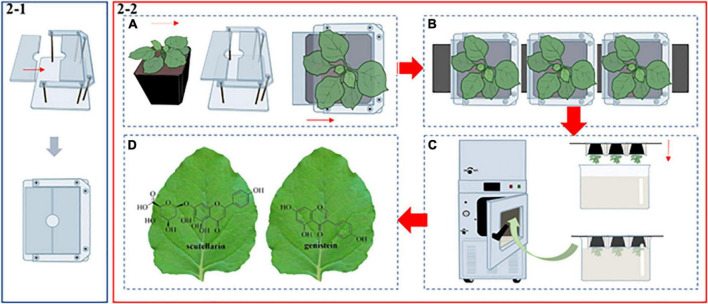
A schematic diagram of the novel E-platform. **(2–1)** The structure of the transparent cube-mold. **(2–2)** Vacuum infiltration process. **(A)** The fabrication process of *N*. *benthamiana*. **(B)** The union of several *N. benthamiana*. **(C)** The leaves were inverted into a stainless steel-tank filled with *A. tumefaciens* suspension. Then, the tank was transferred to the vacuum infiltration device. **(D)** The infiltrated leaves contain two high-value PNPs.

Transient expression by agroinfiltration has been a very mature secondary metabolite production platform. The *N*. *benthamiana* plants used for infiltration experiments were grown for 5 weeks in the soil. As shown in Figure 2-1, a special transparent cube-mold was used to wrap and fix the flowerpot. The mold consists of a removable plastic plate and a shelf. The opening and closing states of the mold can be adjusted while the plastic plate is sliding on the track, and the closed state of the mold will have a hole with a diameter of 0.8 cm. The whole operation process was mainly divided into four parts. First, we placed a single *N*. *benthamiana* into the shelf and pushed the plastic plate to keep the mold closed (Figure 2-2A). Next, we formed a group of three plants and strung them together by threading a flat metal strip across the bottom of the mold (Figure 2-2B). In this case, the fixed flowerpots were inverted into a stainless steel-tank, so that all of the *N*. *benthamiana* leaves could be immersed in a suspension of *A. tumefaciens* (Figure 2-2C). Then, agroinfiltration was carried out in the vacuum infiltration device (Model: DZF-50, Figure 2-2C; Supplementary Figure 2), which was developed by our laboratory (see authors affiliation section) and the Shanghai Wonbio Biotechnology Co., Ltd., Upon starting the device, the vacuum chamber’s pressure dropped to 80 mbar in about 30 s, drawing out the air in the intercellular spaces and allowing the suspension to enter the leaf cells. Finally, the plants were cultured in a dark chamber for 24 h before being brought back to the greenhouse for another 5 days until they were ready to be harvested. The infiltrated leaves were then subjected to the next step of extraction, purification and analysis.

### Color reaction and content determination of flavonoids

**FIGURE 3:**
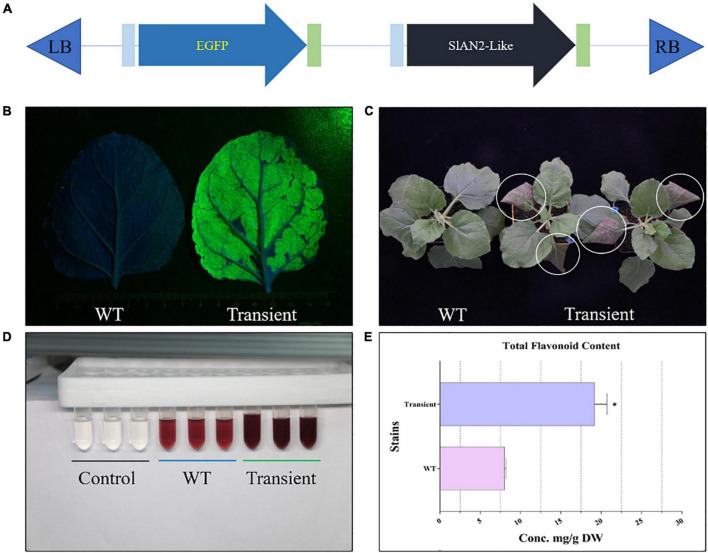
Pre-test of the novel E-platform. **(A)** Schematic diagram of the simple “test unit.” **(B)** GFP fluorescence of the leaves under blue excitation light (approx. 470 nm). **(C)** Phenotypes of WT and infiltrated *N. benthamiana*. **(D,E)** Color reaction and total flavonoid content determination in WT and infiltrated *N*. *benthamiana*. “Transient” represents infiltrated *N*. *benthamiana*. The error bars represent the standard deviations from three independent experiments. The asterisks indicate significant differences (*P < 0.05).

The total flavonoids were extracted as described previously, with some modifications (Zhang et al., 2014). Briefly, leaves of *N*. *benthamiana* were pulverized after being placed in a freeze-dryer for 48 h. Further, 0.02 g of lyophilized powder were incubated in 60% ethanol (20 mL) for 2 h at 60°C with shaking before being centrifuged at 10,000 g for 10 min at 4°C. The aforementioned procedures were repeated again and the supernatants were combined for color reaction and content determination. In this study, flavonoid content was measured using aluminum nitrate in a colorimetric method. In sodium nitrite solution (alkaline conditions), flavonoids react with aluminum salts to form red-orange chelates (Figure 3C), which exhibit a maximum absorption peak of 510 nm. Rutin was used as the standard in this study, and the results were calculated according to the calibration curve (y = 7.6029X-0.0011, R^2^ = 0.9998). See Supplementary Table 2 for detailed operation processes. All measurements were performed in triplicate.

### Liquid chromatograph-mass spectrometer analysis of flavonoids

The total flavonoid content was extracted according to Liu et al. (2019), with a minor modification. Sample powder (0.05 g FW) was sonicated in 1 mL of 100% methanol for 30 min at room temperature. After centrifugation at 12,000 rpm for 10 min, the sample supernatant was filtered through a 0.22 μm syringe filter (Sartorius, Gottingen, Germany) into an UPLC vial. Target flavonoids were detected using an Acquity UPLC I-class/VION IMS QTOF (Waters Corp., Milford, MA, United States) instrument, equipped with a photodiode array detector (PDA). The separation of the target flavonoids was achieved on a C_18_ BEH (1.7 μm, 2.1 × 100 mm) column equipped with a C_18_ BEH (1.7 μm) precolumn (Waters) at 45°C, and the mobile phases consisted of eluent A (0.1% formic acid in Milli-Q water) and eluent B (0.1% formic acid in MeCN, Dingguo Corp., Beijing, China) with a flow rate of 0.4 mL min^–1^. The procedures were as follows: 5–20% B (0–3 min), 20–100% B (3–10 min), 100% B (10–12 min), 100–95% B (12–15 min), 95% B (15–19 min). Finally, 1 μL of the sample was injected into the liquid chromatograph-mass spectrometer (LC-MS) system and the UV wavelength was set at 254 and 335 nm for the detection of genistein and scutellarin. The mass spectrometric analysis and construction of the standard curve were performed according to Xia et al. (2014).

## Results and discussion

### Reconstitution of the genistein and scutellarin synthetic pathways in *Nicotiana benthamiana*

**FIGURE 4:**
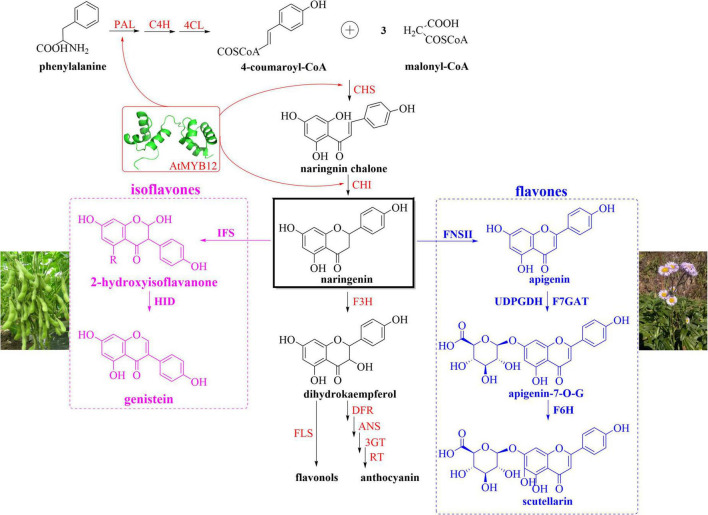
Schematic overview of the genistein and scutellarin biosynthetic pathways in *N*. *benthamiana*. A simplified representation of flavonoids biosynthesis in *N*. *benthamiana* shows key intermediates (black) and enzymes (red). Naringenin (black box), as a crucial node, enters different metabolic pathways catalyzed by different heterologous enzymes. The enzymes in pink box (isoflavones) and blue box (flavones) are derived from soybean and *E. breviscapus*, respectively. The red arrow represents partial genes that can be activated by AtMYB12. PAL, phenylalanine ammonia lyase; CoA, coenzyme A; C4H, cinnamate 4-hydroxylase; 4CL, 4-hydroxycinnamoyl CoA ligase; CHS, chalcone synthase; CHI, chalcone isomerase; IFS, isoflavone synthase; HID, hydroxyisoflavanone dehydratase; F3H, flavanone-3-hydroxylase; DFR, dihydroflavonol reductase; ANS, anthocyanidin synthase; RT, rhamnosyl transferase; 3GT, flavonol-3-glucosyltransferase; FLS, flavonol synthase; apigenin-7-O-G, apigenin-7-O-glucuronide; FNSII, flavone synthase II; UDPGDH, UDP-glucose dehydrogenase; F7GAT, flavonoid-7-O-glucuronosyltransferase; F6H, flavone-6-hydroxylase.

In order to achieve the production of genistein and scutellarin in *N*. *benthamiana*, we analyzed the flavonoid metabolic pathway of *N*. *benthamiana* through the KEGG database (Kyoto Encyclopedia of Genes and Genomes)^1^ and designed the biosynthetic modules as shown in Figure 4. Enzymes and intermediates involved in the flavonoid metabolic pathway of *N*. *benthamiana* were shown in red and black colors, respectively (Figure 4). Based on our understanding of the genistein biosynthetic pathway in leguminous plants such as soybean and kudzu, we screened two crucial genes (*IFS* and *HID*), which have been well identified, characterized and applied (Jung et al., 2000; He et al., 2011; Chu et al., 2014). By overexpressing *IFS* gene, Zhang et al. (2015) and Shih et al. (2008) obtained tomatoes rich in genistein. However, there were also some contrary results for heterologous expression of *IFS*. Suntichaikamolkul et al. (2019) cloned *IFS* from *Pueraria mirifica* and introduced it into *N*. *benthamiana*, which did not produce any isoflavones. Similarly, Shimamura et al. (2007) demonstrated that no isoflavone compounds were accumulated in *Lotus Japonicus* expressing *IFS* alone; what’s interesting was that genistein and daidzein accumulated in large amounts when *HID* was co-expressed. Taken together, the necessity of the *HID* gene for genistein synthesis is controversial. Nevertheless, our previous study also showed that *IFS* expression alone did not lead to the production of genistein. The essential role of 2-hydroxyisoflavanone dehydratase (HID) as a determinant of isoflavone productivity was clearly demonstrated by the result that isoflavones were produced only in the presence of HID. One explanation is that 2-hydroxyisoflavanones (produced by IFS catalysis) trigger negative feedback inhibition of IFS, and that inhibition of IFS is removed by the HID rapidly hydrolyzing 2-hydroxyisoflavanones (Shimamura et al., 2007). Therefore, *IFS* (*GmIFS*) and *HID* (*GmHID*) were added to the isoflavones biosynthetic module (Figure 4, pink box). Scutellarin (breviscapine) is an important flavonoid extracted from *Erigeron breviscapus*, and the synthetic pathway has been deciphered. Liu et al. (2018) identified two key enzymes (F7GAT and F6H) from *E. breviscapus* by genomic analysis and completed biological total synthesis of scutellarin for the first time. As shown in Figure 4, although the biosynthetic pathway from *L*-phenylalanine to naringenin in *N*. *benthamiana* has been well characterized, it does not produce scutellarin due to the lack of *FNSII*, *F6H*, and *F7GAT*. As a consequence, we constructed the flavones synthesis pathway in *N*. *benthamiana* by introducing these three genes (Figure 4, blue box). Among them, FNSII catalyzes the first step of scutellarin biosynthesis. Subsequently, F7GAT functions together with F6H to produce scutellarin from apigenin. In addition to the introduction of genes necessary for the biosynthesis pathway, recent research has underlined the importance of optimizing precursor supply for the production of secondary metabolites in heterologous hosts. AtMYB12 was found to activate multiple genes of the flavonoid metabolic pathway (Figure 4, red arrow represents partial genes), which provided fluxes for the production of genistein and scutellarin. Besides, Zhang et al. (2015) demonstrated that the key genes (*ENO* and *DAHPS*, enolase and 3-deoxy-D-arabinoheptulosonate 7-phosphate synthase) of primary metabolism were significantly activated in *AtMYB12-*overexpressed tomatoes. Moreover, combined expression of *AtMYB12* and stilbene synthase (*StSy*) in tomato increased the concentration of resveratrol and its derivatives to 5–6 mg g^–1^ DW, while in tomatoes expressing *StSy* alone, only small amounts of resveratrol (0.5 mg g^–1^ DW) were found (Zhang et al., 2015). Similarly, Luo et al. (2008) found a 46-fold and an 83-fold increase in endogenous rutin and kaempferol rutinoside levels, respectively, after introducing *AtMYB12* into tobacco. Accordingly, *AtMYB12* was also appended to the biosynthetic modules due to its dual role (Figures 1E, 4).

### General overview of the assembly based on golden gate cloning

GGC provides a precision module-based cloning technique that allows the creation at will and with high efficiency of any eukaryotic multigene construct. In this study, we designed special overhangs to achieve directional assembly of multiple fragments. *Bsa*I recognition sites and specific fusion sites were introduced by primers on both sides of each Level-0 module (Figure 1B and Supplementary Table 3). Additionally, in addition to the use of constitutive CaMV35S-P and NOS-T, we also added the modified 5′-UTR and the 3′-UTR from CPMV RNA2, which dramatically increase protein expression levels (Sainsbury and Lomonossoff, 2008; Liu et al., 2020). In the presence of T4 DNA ligase and *Bsa*I, multiple Level-0 modules were directionally cloned into the destination vectors (Level-1 vectors) (Figure 1C). Furthermore, as shown in Figure 1D, all Level-1 modules contain *Bpi*I recognition sites and special overhangs so that multiple Level-1 vectors containing pathway genes can be directionally integrated into the Level-2 module (pAGM4723). The restriction-ligation was efficient. Engler et al. (2009) achieved 95–100% positive monoclonal results when assembling up to 10 fragments. Based on the GGC, we constructed two independent pathways to produce genistein (Figure 1E, part A) or scutellarin (Figure 1E, part B) in *N*. *benthamiana*. Through the assembly of multi-gene pathways, successful heterologous synthesis of PNPs has been accomplished in recent years thanks to the advancement of synthetic biology. Examples include cannabinoids (*N*. *benthamiana*) (Gülck et al., 2020), CoQ_10_ (*Solanum lycopersicum*) (Fan et al., 2021), nootkatone (*Artemisia annua*) (Gou et al., 2021), taxa-4(5),11(12)-diene (*Physcomitrella patens*) (Anterola et al., 2009), etc. These successful studies further strengthened our confidence in the application of the assembly of multi-gene pathways.

### Production of plant natural products in the *Nicotiana benthamiana* E-platform

Over the past decades, tremendous progress has been made in the production of medicinal proteins and PNPs based on green plants. Unlike the microbial chassis, plant-based production systems are attractive, having a number of advantages such as cost-effectiveness, membrane protein expression, coenzyme and precursor supply, product tolerance, metabolic compartmentalization, and sustainable production (Stephenson et al., 2018), providing a unique model system for the synthesis of medicinal proteins and PNPs. Stable and transient expression systems of host plants are the main production modes. However, the stable expression system has a long cycle and low yield. In recent years, as a rapid and efficient production method, the transient expression system has attracted more and more attention of the scholars. Furthermore, transient expression systems of different plant species have been gradually established. For instance, *Primula vulgaris* (Hayta et al., 2018), *Panicum virgatum* (Chen et al., 2010), *Artemisia annua* (Li et al., 2021), and *Citrus reticulata* (Gong et al., 2021). Compared with these plants, *N*. *benthamiana* is one of the most popular hosts and has been recognized by the scientific community as a green and efficient production chassis. *N*. *benthamiana* is especially well-suited for the synthesis of plant-based PNPs due to its versatile and effective gene transformation system, high quantities of soluble protein, and substantial biomass (up to 100 t ha^–1^) (Tremblay et al., 2010). Although the traditional transient expression systems are widely used, some shortcomings of the system are worth discussing. First, the majority of agroinfiltration procedures are artificially injected with syringes, which damage the leaves and prevent normal growth. In severe cases, foreign genes won’t be expressed normally (Supplementary Figures 3B,E). Moreover, manual injection leads to uneven distribution of *agrobacterium*, which makes it hard to spread to the whole leaf (Supplementary Figure 3D). Furthermore, when handling numerous plant materials, manual injection might waste a lot of time and supplies. Last but not least, some leaves are difficult to inject manually due to their structural properties (e.g., the leaf surface is rich in wax). To address these problems, Osbourn and her team developed a simple vacuum infiltration system (Reed and Osbourn, 2018). On the basis of that system, we improved certain parts. As shown in Figure 2, more leaves can be infiltrated simultaneously by connecting different shelves in tandem in a bigger vacuum chamber. In our study, the device is equipped with two vacuum pumps, which can make the air pressure reach a preset value in a short time. In addition, all parts are controlled by touching the LED “one-touch control” function. We produced two high-value PNPs by combining synthetic biology techniques with this rapid and effective agroinfiltration device (Figure 2-2D). Different from traditional manual injections, the device can automatically complete a large amount of leaf infiltration in a short time, and the post-infiltration leaves are undamaged and filled with *agrobacterium*. However, automated technologies must be incorporated into the system in order to achieve large-scale industrial production.

### The “test unit” verifies the feasibility of the novel E-platform

The synthesis of the target products requires the combination of the multi-gene expression systems and the transformation systems. In order to reduce the time cost caused by the lack of collaboration between the two systems, we designed a simple “test unit” to analyze the feasibility of the novel E-platform from both qualitative and quantitative aspects (Figure 3A). As shown in Figure 3B, *N. benthamiana* leaves with transient expression of “test unit” showed strong green fluorescence under blue light irradiation, which was not detected in the WT, suggesting that the novel E-platform can produce the functional enzymes for the synthesis of target metabolites. Moreover, several TFs have been shown to promote anthocyanidin (flavonoids) accumulation (e.g., *Rosea 1* and *Delila*, *SlMYB75*, *SlAN2-Like*, etc.) (Butelli et al., 2008; Jian et al., 2019). In order to further check whether the two expression cassettes were expressed simultaneously in the E-platform, *SlAN2-Like* was designed into the “test unit.” Phenotypically, “transient” leaves exhibited an intense purple color compared to WT (Figure 3C). Subsequently, the content of flavonoids was further measured. The results showed a 2.41-fold increase in flavonoids content in the “transient” leaves compared to the WT, which was consistent with the results presented in the color reactions (Figures 3D,E). Taken together, the above results indicate that the two target proteins can function simultaneously, revealing the feasibility of the E-platform. Trial-and-error experiments based on this unit will lay the foundation for subsequent experiments and improve the overall work efficiency. Furthermore, the complexity of synthetic biology research dictates the need to engineer trial-and-error experiments, which is also an integral part of the “design-construct-test-learn” closed-loop system advocated by synthetic biology (Clarke and Kitney, 2016).

### Detection of genistein in infiltrated *Nicotiana benthamiana* plants

**FIGURE 5:**
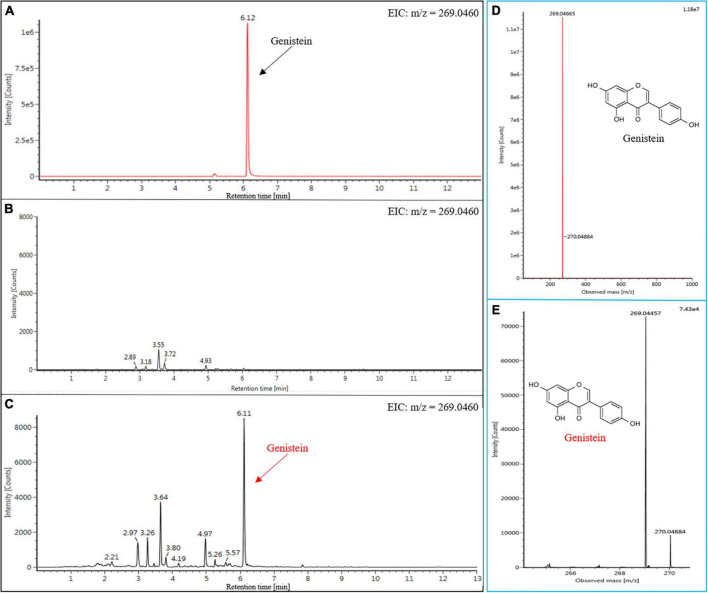
UPLC-MS analysis of genistein in the leaf extracts from infiltrated *N*. *benthamiana* (Detection wavelength, 254 nm). The EIC (*m/z* = 269.0460) of the genistein in the standard sample **(A)**, WT **(B)**, and infiltrated *N*. *benthamiana* **(C)**. MS fragmentation patterns of (-)-genistein in the leaves of infiltrated *N*. *benthamiana* **(E)**, and it was identical to the genistein standard **(D)**.

Metabolic engineering and synthetic biology offer the technical support for the production of genistein on different platforms. Currently, although the microbial platform is still the predominant route for genistein production owing to its high yield (Liu et al., 2022), microbial fermentation requires a constant supply of nutrients, which significantly increases the economic cost. In order to evaluate the production of genistein in *N. benthamiana*, infiltrated and wild type (WT) leaves from *N*. *benthamiana* were analyzed by UPLC-MS to determine their flavonoid profiles. An authentic genistein standard was detected at a retention time of 6.12 min (Figure 5A). The results of the extracted ion chromatogram (EIC) showed the presence of a characteristic peak in the infiltrated leaf samples when compared to WT leaves (Figures 5B,C), which was consistent with that of the authentic genistein standard. Subsequently, we confirmed that the molecular weight of the compound in the new peak was the same as that of genistein by LC-MS (Figures 5D,E; details of the ionic fragments are shown in Supplementary Figure 5). Taken together, these data supported the production of genistein in *N*. *benthamiana*, and the production of genistein via a transient production platform has not been reported previously. Furthermore, the quantitative analysis showed that the approximate content of genistein in infiltrated *N*. *benthamiana* was 5.51 nmol g^–1^ fresh weight (FW), which was higher than that in transgenic *petunia* leaves (3.4 nmol g^–1^ FW) and transgenic *Arabidopsis* leaves (5.4 nmol g^–1^ FW) (Liu et al., 2007). Even so, the current level of genistein is still unsatisfactory. Shih et al. (2008) obtained significant content of genistein (90 nmol g^–1^ FW) in leaves by introducing an isoflavone synthesis pathway into *S*. *lycopersicum*. And beyond that, researchers also synthesized higher levels of genistein in various plant chassis by blocking competitive metabolic branches and/or transferring additional TFs (e.g., *AtMYB12*). Liu et al. (2002) introduced *GmIFS* into *Arabidopsis tt6/tt3* double mutants, leading to the accumulation of much greater amounts of genistein (31–169 nmol g^–1^ FW). Pandey et al. (2014) obtained transgenic tobacco lines by co-expressing *AtMYB12* and *GmIFS*, producing large amounts of genistein in leaves (~219.2 nmol g^–1^ FW). Notably, the use of *AtMYB12* in our study did not enhance the yield of the genistein. Based on the principle of transient protein expression, we speculate that there are two possible reasons for the results. First, the duration of *AtMYB12*’s presence in tobacco cells is unknown, and even some of the proteins are degraded in just a few days, which greatly reduces the effectiveness of *AtMYB12*. Therefore, the difference in sampling time may lead to different results. Second, unlike stable expression systems, transient expression systems have difficulty in ensuring that the target protein (AtMYB12) is expressed in all cells, which results in lower protein expression levels than the former. Nevertheless, the stable expression system still has many drawbacks, such as time-consuming, laborious, and inefficient. In the future, we will continue to optimize and improve the E-platform. Industrial-scale plant factories built on this concept have been used for commercial production of vaccines or natural products. The Medicago plant factory has the capacity to produce 10 million doses of pandemic H5N1 vaccine per month (Lomonossoff and D’Aoust, 2016). Based on this, we can manipulate higher production of PNPs in a short time.

### Scutellarin production in *Nicotiana benthamiana* plants

**FIGURE 6:**
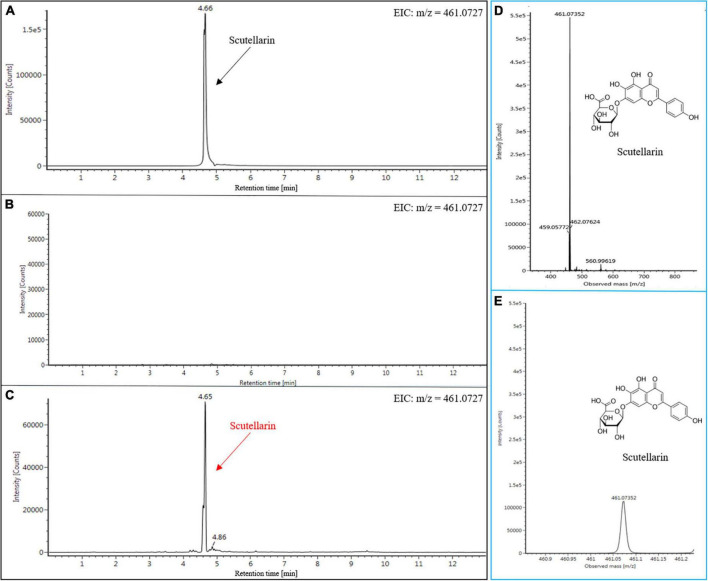
Heterologous production of scutellarin in *N. benthamiana* tissues. EIC (*m/z* = 461.0727) of scutellarin in standard **(A)**, WT **(B)**, and infiltrated *N. benthamiana* tissues **(C)**. MS fragmentation patterns of (-)-scutellarin standard **(D)**. MS fragmentation patterns of (-)-scutellarin in *N. benthamiana* infiltrated tissues **(E)**.

To date, the synthesis of scutellarin has only been reported in yeast (Liu et al., 2018; Wang et al., 2022). Cost-wise, the production of scutellarin through the E-Platform is necessary. In order to assess the scutellarin production in engineered plants of *N. benthamiana*, we used the same extraction and detection approach as for genistein. As expected, scutellarin was detected in *N. benthamiana* leaves after infiltration (Figure 6). The EIC profile of the authentic scutellarin sample shows the presence of a distinct peak (4.66 min) (Figure 6A), which was not found in the leaf extracts from the WT (Figure 6B). However, the result of EIC showed that the characteristic peak (4.66 min) of the extracts from the infiltrated leaves was similar to the authentic scutellarin standard (Figure 6C). The identity of scutellarin was also confirmed by comparing the parent ions and daughter ions of the authentic standards with that of the experimental group (Figures 6D,E and Supplementary Figure 5). Subsequently, we established the standard curve to determine the content of scutellarin (Supplementary Figure 4). The yield of scutellarin in the leaves of infiltrated *N. benthamiana* was 11.35 nmol g^–1^ FW. Besides this, apigenin-7-O-glucuronide (intermediates) was also detected (data not shown), which is also a kind of bioactive compound that possesses remarkable antioxidant, anti-inflammatory, anticarcinogenic, and antispasmodic properties (Wang et al., 2018). To the best of our knowledge, this is the first report on the heterologous biosynthesis of scutellarin in plants. Only Liu et al. (2018) had previously reported synthesizing scutellarin (108 mg L^–1^) in engineering yeast using methods from genomic analysis and synthetic biology. Although scutellarin was successfully synthesized in *N. benthamiana*, its yield was still lower than that of microbial production platforms. The possible reasons for this disparity could be: Firstly, some exogenous proteins may be degraded by the defense system in plant cells, which is also one of the possible reasons why satisfactory yield was not obtained in this study despite the use of AtMYB12. Secondly, the synthesis of target products by plant platforms usually requires substrates present in the plant, which are catalyzed by multiple downstream enzymes at the same time. That’s why the yield of the target products is reduced. In contrast, substrates can be additionally provided in microbial platforms, which is also one of the reasons for the high cost of these platforms. Thirdly, the design route of the target product is relatively independent of the microbial platform, so that the entire metabolic flux can be efficiently utilized. In contrast, the presence of branching enzymes in the plant may break down the metabolic flow and weaken the ability of the plant platform to synthesize the products.

Our work provided a rapid, efficient, and alternative production platform for scutellarin, which can also be applied to the “green production” of other PNPs with high value and low abundance (especially in plants). Furthermore, based on the concept of “smart agriculture” and combined with AI technology, the whole production process will be fully automated. In the future, large-scale transient production platforms will exert greater advantages to contribute to sustainable agricultural development.

## Conclusion

In this study, genistein and scutellarin were produced rapidly and efficiently using the E-platform. Unlike microbial production systems, this novel E-platform requires only sunlight, H_2_O, and CO_2_ to produce the desired products without additional nutrient feeding. Uniquely, this is the first report on the synthesis of scutellarin in a heterologous plant. This work not only demonstrates the power and potential of this novel E-platform, but also provides a reference for the large-scale industrial production of high-value PNPs.

## Data availability statement

The datasets presented in this study can be found in online repositories. The names of the repository/repositories and accession number(s) can be found in the article/Supplementary material.

## Author contributions

KT conceived and supervised the project. KT and XY generated the ideas and performed the experiments. BP designed the device. LL and XY wrote the manuscript. TC, KW, YZ, HL, and XF analyzed the results. All authors revised the results and approved the final manuscript.

## Footnotes

1
https://www.kegg.jp

## Abbreviations

PNPs: plant natural products
CPMV: Cowpea mosaic virus
TFs: transcription factors
UTR: untranslated region
ORF: open reading frame
WT: wild type
LB: Luria-Bertani
KEGG: Kyoto Encyclopedia of Genes and Genomes
LC-MS/MS: liquid chromatography with tandem mass spectrometry
GGC: Golden Gate cloning
AI: artificial intelligence
RS: remote sensing
MeCN: methyl cyanide
DW: dry weight
FW: fresh weight
EIC: extracted ion chromatogram
RPM: revolutions per minute
NOS-T: nopaline synthase terminator
CaMV35S-P: Cauliflower mosaic virus 35S promoter.
