# Supplementary material for: Engineering the expression of plant secondary metabolites-genistein and scutellarin through an efficient transient production platform in Nicotiana benthamiana L
Source: Front Plant Sci. 2022 Sep 6;13:994792. doi: 10.3389/fpls.2022.994792 (PMC9485999; doi:10.3389/fpls.2022.994792)
Supplement: Supplementary file 1 [file Table_1.docx]

**Supplementary Table S1** **The construction steps of multi-gene pathways based on GGC.**

| The assembly process | Step1 | Vectors containing different Level-0 modules were constructed to different Level-1 modules by GGC (**Figure 2B-C**). The following formula was followed (*Bsa*I was used)：  "CaMV35S-P-5'-UTR + AtMYB12 + 3'-UTR-NOS-T + pICH47741"  "CaMV35S-P-5'-UTR + GmIFS1 + 3'-UTR-NOS-T + pICH47751"  "CaMV35S-P-5'-UTR + GmHID + 3'-UTR-NOS-T + pICH47761"  "CaMV35S-P-5'-UTR + EbFNSII + 3'-UTR-NOS-T + pICH47751"  "CaMV35S-P-5'-UTR + EbF6H-2A-EbF7GAT + 3'-UTR-NOS-T + pICH47761" | |
| --- | --- | --- | --- |
|  | Step2 | Construction of Level-2 modules. According to the genes required for metabolite biosynthesis, the Level-1 modules were assembled into 2 artificial transcription units (**Figure 2D-E**). The following formula was followed (*Bpi*I was used)：  Genistein (**Figure 2E, Part A)**  " pICH47732 (NPTII) + pICH47741 (AtMYB12) + pICH47751 (GmIFS1) + pICH47761 (GmHID) + pICH41780 (Linker) + pAGM4723"  Scutellarin (**Figure 2E, Part B)**  " pICH47732 (NPTII) + pICH47741 (AtMYB12) + pICH47751 (EbFNSII) + pICH47761 (EbF6H-2A-EbF7GAT) + pICH41780 (Linker) + pAGM4723" | |
| PCR conditions | 37℃ × 4 min | | 25 cycles |
|  | 16℃ × 4 min | |  |
|  | 37℃ × 30 min | |  |
|  | 65℃ × 10 min | |  |
|  | 70℃ × 5 min | |  |
|  | 4℃ preserve | |  |
