# Supplementary material for: Engineering the expression of plant secondary metabolites-genistein and scutellarin through an efficient transient production platform in Nicotiana benthamiana L
Source: Front Plant Sci. 2022 Sep 6;13:994792. doi: 10.3389/fpls.2022.994792 (PMC9485999; doi:10.3389/fpls.2022.994792)
Supplement: Supplementary file 2 [file Table_2.docx]

**Supplementary Table S2 Summary of flavonoid extraction reagents and procedures.**

| **(mL) groups** | **Blank** | **Measure** | **Standard** |
| --- | --- | --- | --- |
| ddH_2_O | 0.5 |  |  |
| Samples |  | 0.5 |  |
| Different concentrations of rutin solution* |  |  | 0.5 |
| 5% NaNO_2_ solution | 0.03 | 0.03 | 0.03 |
|  | 5 min |  |  |
| 10% Al (NO_3_)_3_ solution | 0.03 | 0.03 | 0.03 |
|  | 5 min |  |  |
| 4% NaOH solution | 0.40 | 0.40 | 0.40 |
| ddH_2_O | 0.04 | 0.04 | 0.04 |
| 15 min △A_510_ = A_Measure_ - A_Blank_ | | | |

* Different concentrations of rutin solution

| Conc. mg/ml | 0 | 0.02 | 0.04 | 0.06 | 0.08 | 0.1 |
| --- | --- | --- | --- | --- | --- | --- |
