# Supplementary material for: Engineering the expression of plant secondary metabolites-genistein and scutellarin through an efficient transient production platform in Nicotiana benthamiana L
Source: Front Plant Sci. 2022 Sep 6;13:994792. doi: 10.3389/fpls.2022.994792 (PMC9485999; doi:10.3389/fpls.2022.994792)
Supplement: Supplementary file 3 [file Table_3.DOCX]

**Supplementary Table S3 Primers used in this study.**

| Primer Name | Sequence (5’-3’) |
| --- | --- |
| GG-35S-F1 | GTCGGTCTCA**GGAG**tgagacttttcaacaaagggt |
| GG-35S-R1 | GTGGGTCTCA**ACAA**tgtcctctccaaatgaaatg |
| GG-5’UTR-F1 | GTCGGTCTCA**TTGT**tattaaaatcttaataggttttgataaaagc |
| GG-5’UTR-R1 | GTGGGTCTCT**AGCG**atcgaatttgggcagaatata |
| GG-35S-F2 | GTCGGTCTCA**GGAG**tgagacttttcaacaaag |
| GG-5’UTR-R2 | GTGGGTCTCA**ACAA**atcgaatttgggcagaatata |
| GG-3’UTR-F | AGCGGTCTCA**CAGC**ttaactctggtttcattaaattttc |
| GG-3’UTR-R | TCAGGTCTCT**AGCG**gatctagtaacatagatgacacc |
| 2A-EbF6H-F | ATCGGTCTCA**TTGT**atggcttctaatgaact |
| 2A-EbF6H-R | CTTGGTCTCA**ATAC**catataaagagaaag |
| 2A-EbF7GAT-F | ATCGGTCTCA**ACCG**atggaaaatattgttgt |
| 2A-EbF7GAT-R | CTTGGTCTCA**GCTG**ttaaactctagc |
| GG-2A-F | AGCGGTCTCA**GTAT**TGGGAAGCGG |
| GG-2A-R | TCAGGTCTCA**CGGT**CCAGGTCCAGG |
| C-AtMYB-12F | TTCTCAAGATGTTTCTGCTGT |
| C-AtMYB-12R | CCATCTCCTCCACAAGTAGTA |
| C-GmIFS1-F | GCTATTAGAAGGCTTACTTATG |
| C-GmIFS1-R | TAGCAAGTGGAACACAAAC |
| C-GmHID-F | AGCTTTTTGTGTTGAATCTGC |
| C-GmHID-R | TGTTCTTCATCTCCAGCATCA |
| C- EbFNSII-F | CCAAATAATAGACTTCCACC |
| C- EbFNSII-R | GCAGTAGTATCAGTAGCAGCA |
| C-EbF7GAT-F | TTTCTTCTACTACTCCATCTA |
| C-EbF7GAT-R | TTAGCATTATCGGAAGTAGCA |
| C-EbF6H-F | ACTTTTAGACTTCATCCACCAA |
| C-EbF6H-R | GATGGATTTTCCCAATGTCT |
| C- p51/61-F | GCACATACAAATGGACGAACG |
| C- p51/61-R | AGTGGTGATTTTGTGCCGAG |
| C-p4723-F | TATGGAAAAACGCCAGCA |
| C-p4723-R | TCTCTTAGGTTTACCCGCCAA |
| C-EGFP-F | ATGGTGAGCAAGGGCGAGG |
| C-EGFP-R | TCACGAAGCCTTGTACAGCT |
| C-SlAN2-Like-F | AAAGAGTTGTAGACTGAGGTGGT |
| C-SlAN2-Like-R | CTTTCAGTGATAATCTTGAGGGT |
| Seq-NPTII-F | GAGATTCCTTGAAGTTGAGTA |
