# Supplementary material for: Engineering the expression of plant secondary metabolites-genistein and scutellarin through an efficient transient production platform in Nicotiana benthamiana L
Source: Front Plant Sci. 2022 Sep 6;13:994792. doi: 10.3389/fpls.2022.994792 (PMC9485999; doi:10.3389/fpls.2022.994792)
Supplement: Supplementary file 4 [file Image_1.pdf]

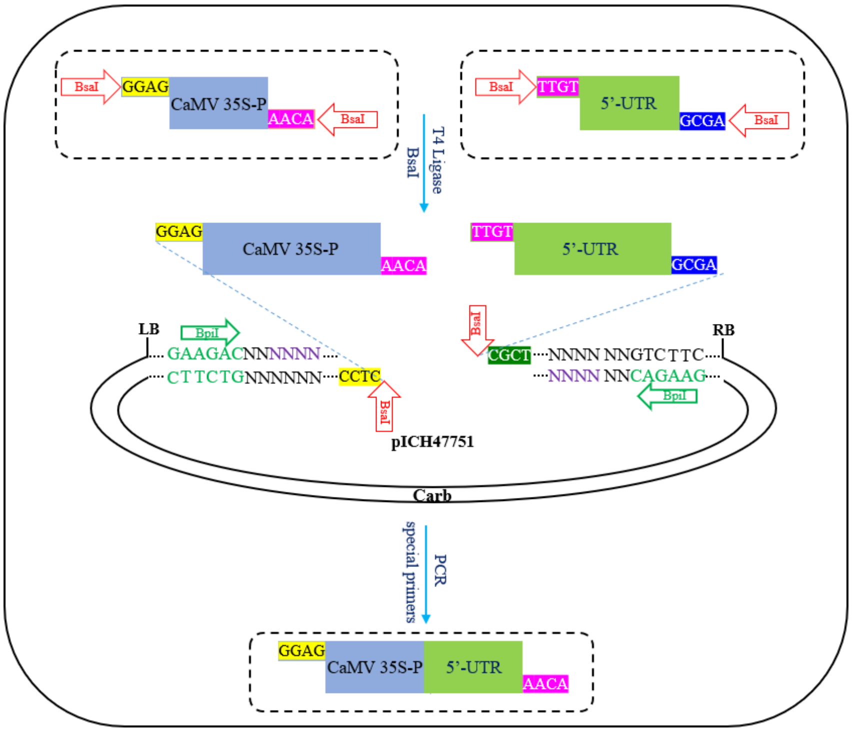

Supplementary Figure S1. Schematic diagram of the assembly process of CaMV35S-P and 5'-UTR. Supplementary Table S3 shows the special primers used in this procedure.
