# Supplementary material for: Engineering the expression of plant secondary metabolites-genistein and scutellarin through an efficient transient production platform in Nicotiana benthamiana L
Source: Front Plant Sci. 2022 Sep 6;13:994792. doi: 10.3389/fpls.2022.994792 (PMC9485999; doi:10.3389/fpls.2022.994792)
Supplement: Supplementary file 5 [file Image_2.pdf]

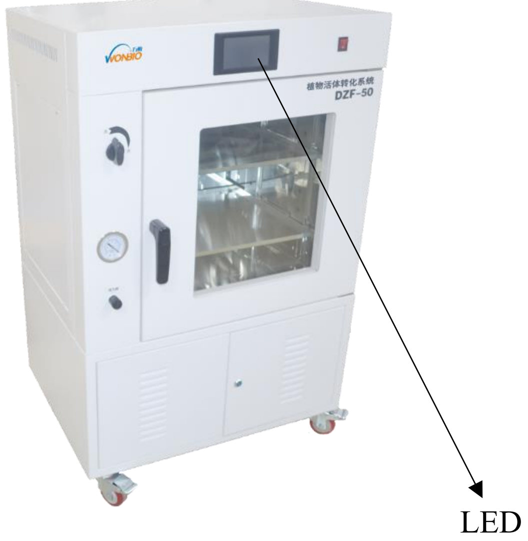

Detailed parameters	
1. Monitor	7-inch LED touch screen
2. Minimal pressure	40 mbar
3. Equipment volume (mm)	510×640×1100 (external); 380×450×400 (inner)
4. Material (vacuum chamber)	Luxurious 304 stainless series
5. Voltage	220 V
6. Weight	145 kg
7. Operating system	Intelligent control systems, automatic control of compressors, solenoid valves, etc.

Supplementary Figure S2. Vacuum infiltration device and parameters.
