# Supplementary material for: Engineering the expression of plant secondary metabolites-genistein and scutellarin through an efficient transient production platform in Nicotiana benthamiana L
Source: Front Plant Sci. 2022 Sep 6;13:994792. doi: 10.3389/fpls.2022.994792 (PMC9485999; doi:10.3389/fpls.2022.994792)
Supplement: Supplementary file 6 [file Image_3.pdf]

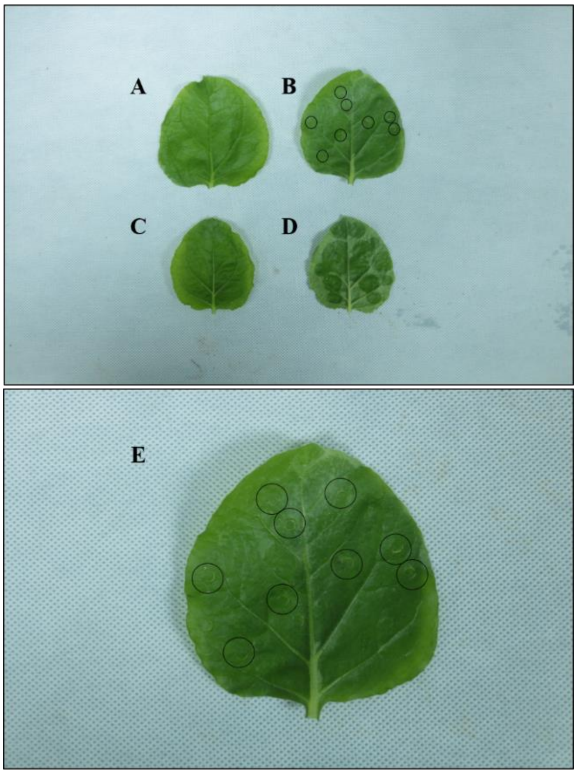

Supplementary Figure S3. Manual injection (A and C) and vacuum infiltration (B and D). The black circles represent areas of leaf damage. E, Enlarged image of Figure B.
