# Supplementary figures and images for: Engineering the expression of plant secondary metabolites-genistein and scutellarin through an efficient transient production platform in Nicotiana benthamiana L
Source: Front Plant Sci. 2022 Sep 6;13:994792. doi: 10.3389/fpls.2022.994792 (PMC9485999; doi:10.3389/fpls.2022.994792)

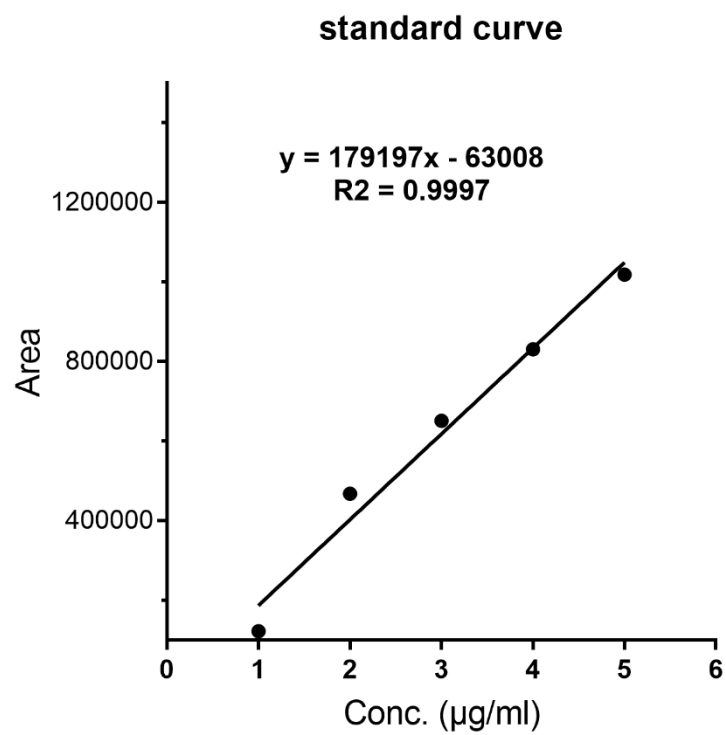

**Supplementary Figure S4. Establishment of a scutellarin standard curve.**

Supplement: Supplementary file 7 [file Image_4.pdf]
