# Supplementary material for: Engineering the expression of plant secondary metabolites-genistein and scutellarin through an efficient transient production platform in Nicotiana benthamiana L
Source: Front Plant Sci. 2022 Sep 6;13:994792. doi: 10.3389/fpls.2022.994792 (PMC9485999; doi:10.3389/fpls.2022.994792)
Supplement: Supplementary file 8 [file Image_5.pdf]

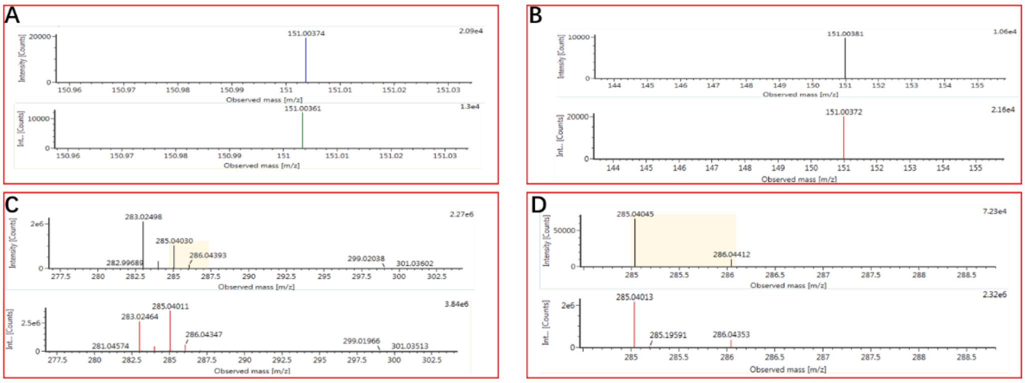

Supplementary Figure S5. The daughter ions of the authentic standards and the experimental group. A and B, authentic genistein standards and the products. C and D, authentic scutellarin standards and the products.
